# Synthesis, Characterisation, Photocatalytic Activity, and Aquatic Toxicity Evaluation of TiO_2_ Nanoparticles

**DOI:** 10.3390/nano11123197

**Published:** 2021-11-25

**Authors:** Luminita Andronic, Alina Vladescu, Alexandru Enesca

**Affiliations:** 1 Product Design, Mechatronics and Environment Department, Transilvania University of Brasov, Eroilor 29, 500036 Brasov, Romania; a.enesca@unitbv.ro; 2 National Institute of Research and Development for Optoelectronics INOE2000, 409 Atomistilor St., 77125 Magurele, Romania; alinava@inoe.ro; 3 Physical Materials Science and Composite Materials Centre, Research School of Chemistry & Applied Biomedical Sciences, National Research Tomsk Polytechnic University, 30 Lenina Avenue, 634050 Tomsk, Russia

**Keywords:** titanium oxide, photocatalysis, imidacloprid, scavengers, aquatic toxicity, *Chlorella vulgaris*

## Abstract

Imidacloprid (IMD) is a toxic pesticide, and is one of the eight most widely used pesticides globally. Heterogeneous photocatalysis has often been investigated in recent years and can be successfully applied to remove imidacloprid from water. However, less investigated is the toxic effect of both the photocatalyst and the pesticide on aquatic life. Titanium dioxide (TiO_2_) remains the most effective photocatalyst, provided it is not toxic to the aquatic environment. This study investigated the TiO_2_ synthesis, characterisation, and photocatalytic activity on imidacloprid degradation and the toxicity of TiO_2_ nanoparticles and imidacloprid on the green algae *Chlorella vulgaris*. In the photodegradation process of IMD (initial concentration of 20 mg/L), electrons play an essential role; the degradation efficiency of IMD after 6 h increased from 69 to 90% under UV irradiation when holes (h^+^) scavengers were added, which allowed the electrons to react with the pollutant, resulting in lowering the recombination rate of electron-hole charge carriers. Growth inhibition of *Chlorella vulgaris* and effective concentration (EC50) were determined to study the toxic effect of TiO_2_ nanoparticles and imidacloprid. The EC50 increased from 289.338 mg/L in the first 24 h to 1126.75 mg/L after 96 h *Chlorella vulgaris* algal age, when the toxicant was TiO_2_. When IMD was the aquatic toxicant, a decrease in EC50 was observed from 22.8 mg/L (24 h) to 0.00777 mg/L (120 h), suggesting a long-term high toxicity level when pesticides in low concentrations are present in an aquatic environment.

## 1. Introduction

Nowadays, more and more pesticides are used to control pests, especially in agriculture, representing an important class of emerging pollutants present in water [1]. The most widely used pesticides in the world are acetamiprid, clothianidin, dinotefuran, imidacloprid, nitenpyram, and thiacloprid [2,3]. Imidacloprid is a relatively broad-spectrum systemic insecticide that is water soluble compared to other non-polar insecticides [4]. It is used as a foliar treatment in various crops against insects. Its persistence in an aqueous medium is 41.6 days at pH = 9 and 36.2 days at acidic pH [5]. However, imidacloprid enters the groundwater by spray drift or drainage after application and harms aquatic life and human health [6], and a residual concentration in the water has been reported to cause adverse effects to the aquatic environment [7].

Pesticides are highly toxic in the aquatic environment in very low concentrations of the order of µg/L up to 20 mg/L, and for their elimination from water, heterogeneous photocatalysis is a solution that can lead to their complete mineralisation [8]. Besides a detailed study of the mechanism of photodegradation and mineralisation, which have been intensely investigated in recent years, the toxicity of pesticides and photocatalysts on the aquatic environment is a subject that has been poorly investigated [9]. The involvement of reactive oxygen species (ROS) resulting from the irradiation of semiconductors in photocatalysis processes can be highlighted by the reaction of ionic species with scavengers [10]. Reactive oxygen species (ROS) are free radicals, molecules, or ions activated by energy adsorption and produced in chemical reactions [11]. The most important ROS results in the photodegradation processes of water pollutants are: hydroxyl radicals (${\mathrm{HO}}^{\cdot }$), superoxide anion ($O_{2}^{\cdot -}$), and hydrogen peroxide molecules (H_2_O_2_) [12]. ROS generation and release during photocatalytic processes in the presence of scavengers can cause cell destruction of green algae [13]. ROS at a low concentration can function as redox messengers, playing a significant role in intracellular processes [14]. Exceeding the physiological concentration causes these radicals to become oxidative stress and cause cell damage and cell death [15]. Effective concentration (EC50) is an index used in the toxicological assessment of compounds calculated based on specific growth rate inhibition [16]. For example, EC50 in alga, the growth inhibition test (OECD) [17] is the concentration of the test compound, resulting in a 50% reduction in either growth or growth rate relative to the control. According to EU-Directive 93/67/EEC, EC50 is used to classify compounds into different toxicity classes: EC50-values <1 mg L^−1^ (very toxic to aquatic organisms); 1–10 mg L^−1^ (toxic to aquatic organisms); 10–100 mg L^−1^ (harmful to aquatic organisms); and >100 mg L^−1^ (not classified) [18].

The objectives of the present research are: (1) to synthesise and characterise TiO_2_ nanoparticles with different ratios between anatase and rutile; (2) evaluation of the photocatalytic activity of nanoparticles and the reactive oxygen species (ROS) study that defines the photocatalysis mechanism; and (3) to determine the toxicity of the photocatalyst nanoparticles and wastewater pollutant against algal growth by exposing *Chlorella vulgaris* at the exponential growth phase to TiO_2_ nanoparticles and imidacloprid, a pesticide chosen as a pollutant, and measuring the growth inhibition rate against the control. Solving the three objectives will lead to the correct evaluation of photocatalysis, and a green and clean environment.

## 2. Materials and Methods

### 2.1. Photocatalysts Synthesis

The precursors used to obtain TiO_2_ powder by the sol–gel method were: titanium isopropoxide (IV) (Ti[OCH(CH_3_)_2_]_4_, TTIP, 97%, Alpha Aesar) used as the precursor of titanium, ethanol, and nitric acid. Mix TTIP with ethanol in a 1:1 volumetric ratio, homogenise by stirring for 30 min, and dropwise add a 1 M HNO_3_ solution to a sol, stirring for 2 h. Allow 24 h to form the TiO_2_ gel and leave the gel in the oven at 110 °C to obtain the dry gel. The titanium dioxide gel is amorphous, so heat treatment is required to obtain crystalline titanium dioxide. The sample of TiO_2_ formed was heat-treated for 3 h at various temperatures, respectively 400, 500, 600, 700, and 800 °C in a 0.5 atm argon flow.

### 2.2. Photocatalysts Characterisation

The phase composition, crystallinity, texture, and crystallite size were analysed by the X-ray diffraction method (XRD) (SmartLab diffractometer, Rigaku, Tokyo, Japan), with a Cu target X-ray tube (λ = 0.15405 nm). The measurements were taken from 20 to 100°, at a step size of 0.02°. Identification of peaks was performed by rutile card no. 01-076-1941 and anatase card no. 021-1272. The microscopic morphology of the samples was obtained using a scanning electron microscope (SEM) (Hitachi TM3030 Plus, Tokyo, Japan) and an energy dispersive X-ray spectrometer (EDX) (Bruker, MA, USA) for elemental analysis of the surface.

The values for the direct band-gap were determined by the UV–Vis diffuse reflectance spectra using the Kubelka–Munk transformation. The band-gap energies were estimated from the Tauc plots of [F(R)hν]^2^ vs. photon energy (hν), where the intercept of the tangent to the *x*-axis gives the E_g_ values for a direct allowed transition.

The textural properties were determined by Brunauer–Emmett–Teller (BET) adsorption–desorption of N_2_ at −196 °C with a pre-treatment of degassing for four hours at 250 °C under vacuum (10^−3^ Pa). The following parameters were determined: (i) the total pore volume (Vt) at a relative pressure of 0.95 using the non-local density functional theory (NLDFT) model; (ii) the BET-specific surface area; (iii) the micropore volume, micropore area, small micropore volume (p/p_0_ = 0.25–0.8), large micropore volume (p/p_0_ = 0.8–0.95) by t-plot; and (iv) total mesopore volume (DJH) (2.0–50 nm) were analysed using the Barrett–Joyner–Halenda (BJH) model. In addition, the micropore size and nanoparticle size were determined.

The isoelectric point of a material called the point of zero charge—pH_zpc_ is defined as the pH at which the material’s surface is electrically neutral. Thus, the material surface loading is positive (Equation (1)) at pH values below values recorded for pH_zpc_ and negative for pH values above pH_zpc_ (Equation (2)), respectively [19].

(1) $${\mathrm{Ti}}^{\mathrm{IV}}-\mathrm{OH}+H^{+}\to {\mathrm{Ti}}^{\mathrm{IV}}-{\mathrm{OH}}_{2}^{+}$$

(2) $${\mathrm{Ti}}^{\mathrm{IV}}-\mathrm{OH}+{\mathrm{OH}}^{-}\to {\mathrm{Ti}}^{\mathrm{IV}}-O^{-}+H_{2}O$$

The isoelectric point of a material is dependent on the properties of the material and the environmental conditions in which it is found, and it is essential to evaluate pH_zpc_ in the choice of photodegradation parameters due to the surface charge of the photocatalyst surface concerning the molecular structure of the degraded organic pollutant. By choosing a pH range favourable to the electrostatic attraction between the photocatalyst and the pollutant, increased efficiency of the photodegradation process can be ensured [20]. The determination of the isoelectric point was experimentally conducted by acid–base titration. The 0.6 g/L powder was dispersed in 50 mL of 0.1 M NaOH solution and titrated with 0.1 M HCl solution recording the variation in pH (Hanna edge HI 98194) with the volume of the acid solution added. The pH determined according to the volume of added HCl was plotted, the inflexion point being the isoelectric point. For its exact determination, the graph derived from the first order of pH was plotted according to the volume of HCl added. The maximum of the most significant peak represents the volume of equivalence that corresponds to the isoelectric point.

### 2.3. Photocatalytic Tests

The degradation of imidacloprid (IMD) was performed in a homemade reactor equipped with blacklight tubes (180 W) emitting UV radiation centred at 365 nm and visible simulated irradiation (180 W VIS light tubes and 108 W, UV blacklight tubes). Aqueous solutions of imidacloprid were prepared daily in double deionised water in concentrations between 5 and 40 ppm with a natural pH of around 6.35. The TiO_2_ powder optimised (0.6 g/L) was mixed with the IMD solution for 15 min in the dark to reach the adsorption–desorption equilibrium. The samples were irradiated for fixed periods. During the photocatalytic reaction, the aliquots of 1.35 mL were taken periodically (0…360 min), and 0.15 mL of acetonitrile was added to desorb the IMD from the photocatalyst surface. The mixture was filtered by a 0.45 µm Millipore PES filter and analysed with a HPLC–DAD (UV–VIS) analyser. HPLC analysed aqueous solutions of imidacloprid, consisting of a Shimadzu LC-20 ADsp chromatograph, equipped with a Nucleosil C18-Macherey Nagel, Nucleosil^®^ 5 µm C18 100 A, LC Column 250 × 4.6 mm, and a UV detector SPD-20 A at an absorption wavelength of 270 nm. The column thermostat was maintained at 45 °C. The mobile phase was a mixture of 70%v acetonitrile and 30%v ultrapure water—the flow rate was 1.2 mL min^−1^ with a 10 μL sample injection volume. The retention time for IMD was 2.76 min. For quantification purposes, calibration curves in the range from 0.5 to 40 mg L^−1^ were validated, using nine concentration levels with correlation coefficient R^2^ = 0.9996. The limit of detection (LOD) and the limit of quantification (LOQ) of the imidacloprid were 0.7848 and 2.378 mg·L^−1^, respectively. The residual concentration of IMD was measured after t minutes. The degradation rate of IMD was calculated as the degradation efficiency using Equation (3):

(3) $$\eta =\frac{c_{0}-c}{c_{0}}\cdot 100$$

where c_0_ is the initial concentration of IMD solutions (ppm), and c is the concentration of IMD solutions (ppm) at a time t [20].

Reactive oxygen species (ROS) such as superoxide radicals ($O_{2}^{\cdot -}$), hydroxyl radicals (${\mathrm{HO}}^{\cdot }$), electrons ($e^{-}),$ and holes ($h^{+}$) are responsible for choosing the photodegradation mechanism. A total of 1 mM 1,4 benzoquinone was used to trap superoxide radicals, 1 M isopropyl alcohol as the hydroxyl radical scavenger, 20 mM formic acid as the positive hole scavenger, and 1 mM K_2_Cr_2_O_7_ as the electron scavenger. In addition, the kinetics of imidacloprid was investigated considering P500 photocatalytic materials.

### 2.4. Toxicity Effect of TiO_2_ Nanoparticles and Imidacloprid on Chlorella vulgaris Growth

The toxicity of the TiO_2_ photocatalyst (sample P500) and imidacloprid as wastewater pollutants were tested as toxic compounds that affect green algae growth. *Chlorella vulgaris* (CCAP211/11B) was provided from Culture Collection Algae and Protozoa (CCAP, Scottish Marine Institute, Dunberg, Scotland) and acclimatised to our laboratory conditions.

*Chlorella vulgaris* were cultured in freshly prepared Bold Basal medium (3N-BBM + V), a ratio of culture medium, and algae of 1:100 were chosen for the tests with a 16 h:8 h light–dark cycle (temperature around 25.0 °C) [21]. Cultures were irradiated with four tubes of fluorescent F18/T8 Sylvania aquastar (18 W, 900 lux each) (16 h:8 h light:dark controlled by a timer), were permanently aerated with a pump with a flow rate of 3 L/min. The photosynthetically active radiation (PAR) was approximately 50 μmol m^−2^ s^−1^ measured with the quantum sensor LI-190R-BNC-2, attached to the LI-250 A light meter (LI−COR Bioscience GmbH Homburg, Germany).

The exponential phase cultures of *Chlorella vulgaris* that contained 1 × 10^5^ cells·mL^−1^ in 250 mL Bold Basal medium was exposed to TiO_2_ (0.075, 0.15, 0.3, 0.6 and 1.2 g·L^−1^, respectively) and imidacloprid (5, 10, 20, 30 and 40 mg·L^−1^, respectively) in a beaker under 16 h:8 h light:dark, photon concentration: 50 μmol·m^−2^·s ^−1^ and static condition for 120 h. The samples were agitated every 24 h to avoid the settling of algal cells. The concentrations of TiO_2_ and imidacloprid were selected based on the concentration used in the photocatalysis experiments. *Chlorella vulgaris* cell suspension without exposure to contaminants under the same experimental conditions was considered as the control. Measurements were made in triplicate for each experiment. The results reported were the average of all replicates. At least one control group with no addition of nanoparticles was carried out in parallel in each replicate.

Algae growth control was performed by counting the cell numbers by loading 10 µL of algal cell suspension into a Neubauer counting chamber (Marienfeld) placed under an optical microscope (Bresser, Germany). The growth of the algae culture was monitored every 24 h up to 120 h. The growth inhibition (μ%) was calculated based on Equation (4), given below.

(4) $$\mu =\frac{\mathrm{lnN}-{\mathrm{lnN}}_{0}}{t}$$

where μ is the specific growth rate (day^−1^); N is cell numbers counted every 24 h during the exposure period (120 h); N_0_ is cell counting in a control culture, without TiO_2_ and IMD; and t = exposure time (day).

The toxicity tests were developed according to the International Standard ISO 8692:2012 [22], ISO 14442 [23], and Test No. 201 [17] for green algae, respectively. Growth inhibition and effective concentration were determined. The effective concentration of TiO_2_ and imidacloprid, which produced a 50% (EC50) respectively 90% (EC90) inhibition of algal growth to 24 h until 120 h exposed to the nanoparticles or pesticides, was obtained by linear regression of specific growth rate inhibition vs. logarithmic concentrations. Experimental data were analysed with Origin Pro 9.

## 3. Results and Discussion

### 3.1. Materials Characterisation

**Figure 1:**
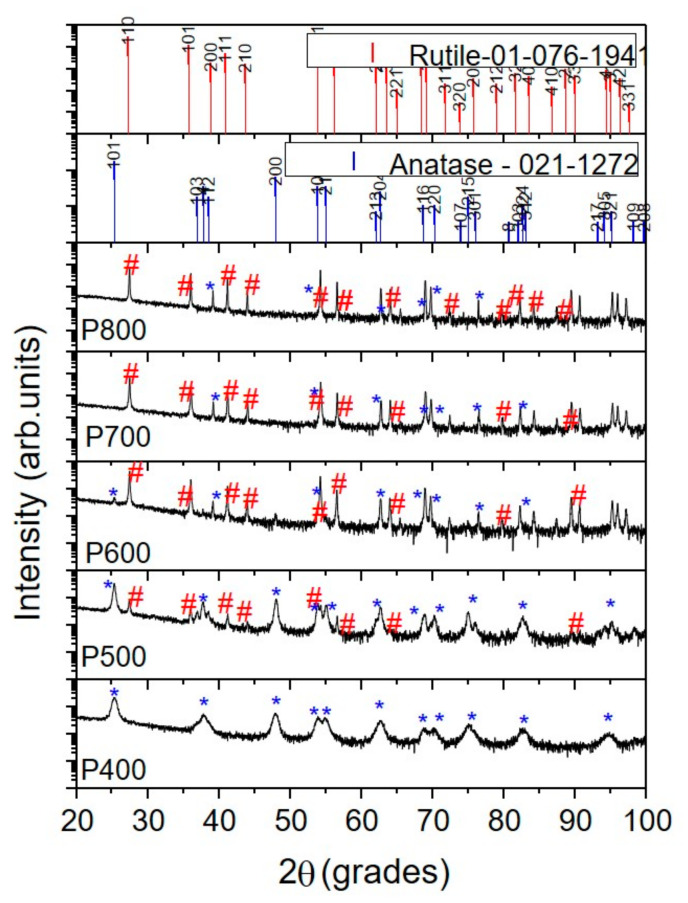
The XRD diffraction patterns of the investigated samples (* is anatase phase # is rutile phase).

XRD diffraction patterns are presented in Figure 1. The X-ray diffraction identifies phases and the value of the degree of crystallinity (Table 1). The crystallite size was calculated from the XRD pattern, according to the highest intensity peak corresponding to the (101) plane for anatase and (110) plane for rutile using the Debye–Scherrer equation: d = k λ/β·cosθ, where d is the crystallite size, λ is X-ray wavelength (1.54178 Å, θ is the diffraction angle, and β is full width at half maximum (FWHM), the results of which are shown in Table 1. It can be observed that crystallinity and the anatase particle increase with increasing calcination temperature from 400 to 600 °C (Table 1). The rutile crystallite size decreased from 72.5 to 10.3 nm with an increase in the temperature from 600 to 800 °C.

The texture coefficient T(hkl) was calculated using Equation (5).

(5) $$T\left(\mathrm{hkl}\right)=\frac{I\left(\mathrm{hkl}\right)}{\sum I\left(\mathrm{hkl}\right)}$$

where I(hkl) is the intensity of the line (hkl) and ΣI(hkl) is the sum of the intensities of all detected diffraction peaks.

The texture coefficients calculated on the most important detected peak of each phase of TiO_2_ (anatase or rutile) can be found in Table 2. According to the values presented in Table 2, one may observe that samples P400 and P500 were formed mainly of the anatase phase and textured in the (101) direction, while the P600, P700, and P800 samples exhibited a strong (110) preferred orientation of the rutile phase.

Both anatase and rutile phases were tetragonal in structure. According to rutile card no. 01-076-1941, the cell parameters were a = 4.6230 Å and c = 2.9860 Å, while for the anatase card no. 021-1272, a = 3.7852 Å and c = 9.5139 Å. The investigated samples exhibited the following cell parameters:

P400: anatase a = 3.7905 ± 0.0021 Å and c = 9.4924 ± 0.0108 Å.

P500: anatase: a = 3.7892 ± 0.0114 Å and c = 9.4032 ± 0.0614 Å; rutile: a = 4.7382 ± 0.0371 Å and c = 2.8630 ± 0.0418 Å.

P600: anatase: a = 3.7916 ± 0.0077 nm and c = 9.0672 ± 0.0436 nm; rutile: a = 4.5374 ± 0.1598 and c = 2.9765 ± 0.1499 nm.

P700: rutile: a = 4.5872 ± 0.0121 Å and c = 2.9411 ± 0.0092 Å.

P800: rutile: a = 4.5749 ± 0.1305 Å and c = 2.9653 ± 0.1316 Å.

Comparing the values of the “a” cell parameter of the rutile phase, the P500 has valued slightly higher than that of other samples. For the P600, P700, and P800 samples, the “a” value decreased.

Regarding the values of the “a” cell parameter of the anatase phase, a nearly random distribution could be observed, while the “c” parameter decreased compared with the value of the anatase card, indicating compressive residual stress. All these findings revealed that the lattice distortion takes place due to various effects. One of these is the growth of rutile crystallites as an overlayer of rutile on anatase particles and how it was reported by Bickley et al. [24]. Another reason, rutile could form in the bulk of the anatase grains, leaving a surface layer of anatase on the rutile particles [25]. Moreover, the decrease in the lattice distortion may signify the presence of dopants and/or impurities, mainly if these dopants are preferentially present in one of the phases.

**Figure 2:**
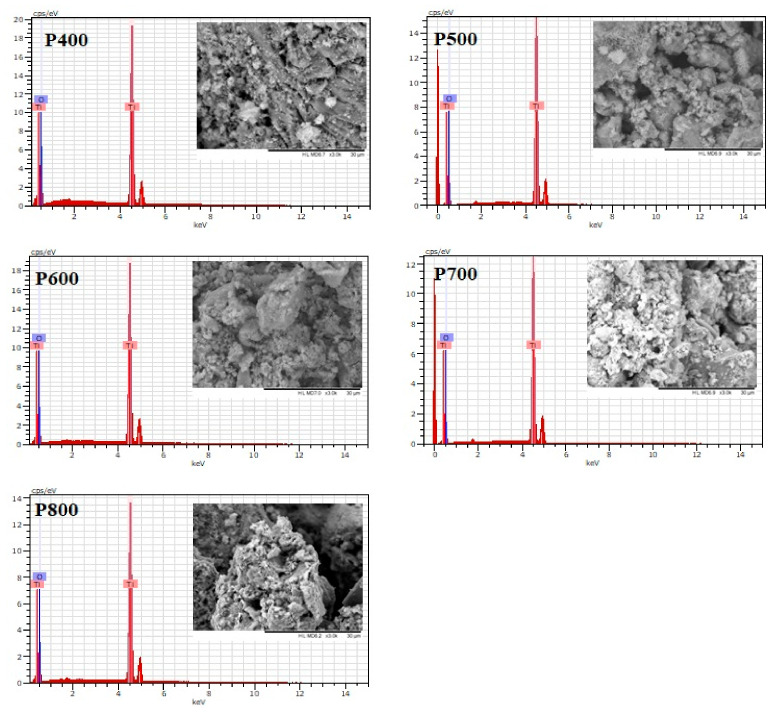
SEM morphology (scale bar = 30 µm) and EDX composition for the TiO_2_ nanoparticles.

The SEM morphology, together with the chemical composition investigated with EDX, is shown in Figure 2. The TiO_2_ particles exhibited irregular porous morphology due to a large agglomeration of nanoparticles at all temperatures without significant changes in the analysed samples. In order to clarify the nanoparticles’ elemental composition, the EDX analysis was conducted on the surface of the nanoparticles during SEM observations. As a result, only titanium (Ti) and oxygen (O) were detected on the samples’ surface, proving no other peak as observed in the spectra, concluding that no other metallic impurities were detected from the precursors.

The nanoparticles’ photocatalytic properties can be studied by the materials’ band-gap, which determines how many photons are adsorbed by the semiconductor electrons to transit from the valence band (BV) to the conduction band (BC). The values of the band-gap energy for the synthesised samples are given in Table S1. E_g_ values observed at around 3.2 eV were characteristic of the anatase crystalline structure and values of 2.93 and 2.96 eV specific to the rutile crystalline structure, structures confirmed by XRD analyses (Figure 1), which could enhance photocatalytic activity at higher wavelengths (under visible light irradiation). The samples treated at higher temperature showed a band-gap reduction.

The specific surface area plays an important role in the photocatalytic activity of materials. The specific surface area of the powders was determined and calculated using the Brunauer–Emmett–Teller (BET) method determined from the relative pressure range between 0.05 and 0.30. The pore size distribution and pore volumes were calculated with desorption data from adsorption–desorption isotherms based on the Barrett–Joyner–Halenda theory (BJH); the results are shown in Table S2. The measured values of pore size, pore volume, surface area, nanoparticle size, and BET surface area are presented in Table S2, considering mesopores (2–50 nm), with a distinction between small mesopores (2–10 nm) and large mesopores (10–50 nm). As evident in Figure S1, the synthesised TiO_2_ displayed type IV and type III adsorption isotherms, with a clear distinction in the type of hysteresis loop on post calcination temperature: H2 for 400 °C, H3 for 500 °C and H1 for 600, 700, and 800 °C. The calcinated samples at higher temperatures (600–800 °C) exhibited an H1 type hysteresis loop with a decrease in the pore diameter, pore volume, and surface area, and increase in nanoparticle size, as shown in Table S2, with a direct consequence on the photocatalytic activity of the materials. The sample P400 and P500 displayed a narrow pore size distribution centred at 7.32 and 8.99 nm and a decrease in measured surface area from 89.08 to 34.69 m^2^·g^−1^, respectively. The increase in heat treatment temperature led to the decrease in the specific surface of the materials from 89.08 to 3.12 m^2^·g^−1^, contributing to the reduction in nanoparticle photocatalytic activity.

The nanoparticles’ point of zero charge (pH_ZPC_) was calculated by plotting V_total_ as a pH function and obtaining the first-order derivative (Figure S2). Values around 6 … 6.5 were very close to 6.35 as the pH of the IMD solution at which the photocatalysis takes place. The values calculated indicate that IMD can be adsorbed on the surface of nanoparticles in both forms, molecules or ions.

### 3.2. Evaluation of the Photocatalytic Activity and Kinetics of TiO_2_ on Imidacloprid Degradation

The photocatalytic performance of the TiO_2_ series was evaluated by IMD degradation under different light source irradiation (UV and simulated visible light such as a combination between UV and VIS irradiation) in the neutral medium. Recently, Garg and Derbalah observed in their studies that imidacloprid degradation was more effective at neutral pH than in basic or acidic conditions [26,27]; the photodegradation experiments were conducted under neutral conditions. 

The equilibrium concentration of imidacloprid evaluated after 30 min in the dark was negligible, and was used in the photocatalysis evaluation instead of the initial concentration. Imidacloprid photolysis in the absence of TiO_2_ photocatalyst under simulated sunlight conditions (λ = 300–700 nm) using UVA lamps (λ = 365 nm) and artificial lamps showed negligible influence.

**Figure 3:**
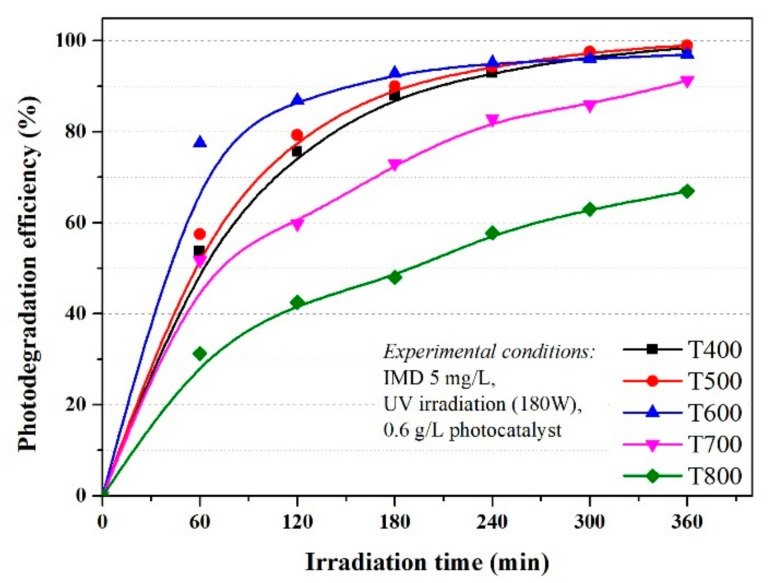
The influence of photocatalysts and UV radiation on the photodegradation efficiency of IMD (with an initial concentration of 5 ppm).

**Figure 4:**
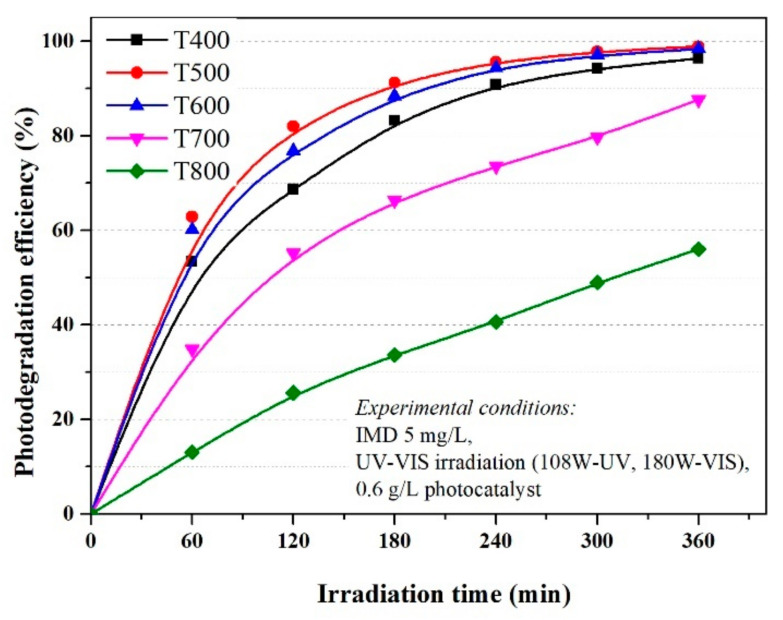
The influence of photocatalysts and UV–Vis radiation on the photodegradation efficiency of IMD (with an initial concentration of 5 ppm).

The degradation efficiency of imidacloprid increased in the first 120 min at efficiencies of over 80% for materials in which the anatase crystalline form predominates (P400 and P500), then reached 99% at 300 min, similar for UV (Figure 3) and simulated visible light (Figure 4). Although rutile has a weaker photocatalytic activity than anatase, as stated in the literature, the increase in efficiency is sudden in the first 60 min, then linear up to 360 min when it reaches efficiencies of 91% and 67% for P700 and P800, respectively, in conditions of UV irradiation, which was slightly lower under simulated visible light irradiation, but with a similar mechanism. Thus, the optimum reaction time was 120 min.

As shown in Figure 3 and Figure 4, the photocatalytic activities of the photocatalysts were higher with similar efficiency under UVA irradiation and simulated visible light, with an imidacloprid degradation efficiency between 96 and 99% for samples T400, T500, and T600, after 360 min. The results were also good for sample T700, with an efficiency of 91% under UVA irradiation and 88% under simulated visible light. The lowest efficiency was only 67% in the T800 sample under UVA and 56% under simulated visible light after 360 min. This decrease compared to the other samples was due to the smallest particle sizes of only 10 nm (obtained from XRD data). With only rutile in the sample, the lowest value of thee BET surface area was only 3.1262 m^2^/g, and last but not least, the agglomeration particles observed in the SEM and porosity analysis.

Photocatalytic degradation of imidacloprid using TiO_2_ is well reported in the scientific literature and explains the first-order degradation kinetics and the study of degradation products [26,27,28]. The photodegradation behaviour can be described as first-order Langmuir–Hinshelwood kinetics (Equation (6)), where *C*_0_ is the initial concentration of IMD, *C* is the concentration of IMD at time t, and k is the reaction rate constant [29]. The reaction rate constants resulted from the slopes of each fitting line (Equation (7)). The first-order kinetic constants estimated within our work differed from other photocatalytic studies due to the different experimental setup and conditions such as the amount of catalyst, pH, pollutant volume and initial concentration, the geometry of the reactor, and photon flux. In our case, it must be clearly stated that the imidacloprid degradation has replicable results under the same experimental conditions.

(6) $$\ln\left(\frac{C}{C_{0}}\right)=k\cdot t$$

(7) $$\ln\frac{C}{C_{0}}=f\left(t\right)$$

**Figure 5:**
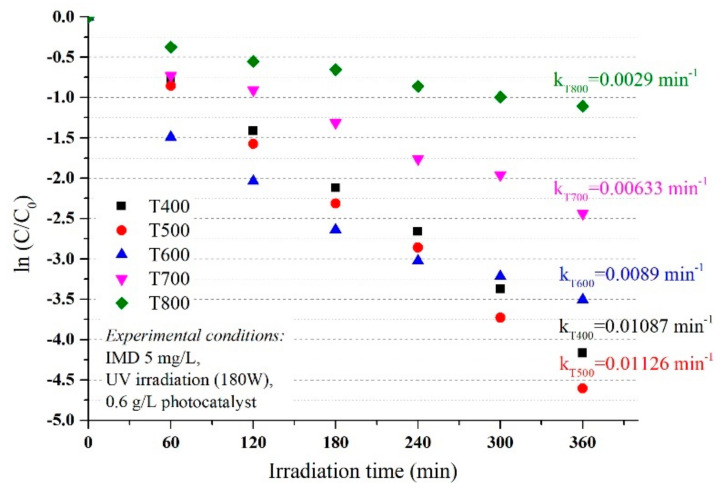
The degradation kinetics vs. catalyst type under UV irradiation conditions.

**Figure 6:**
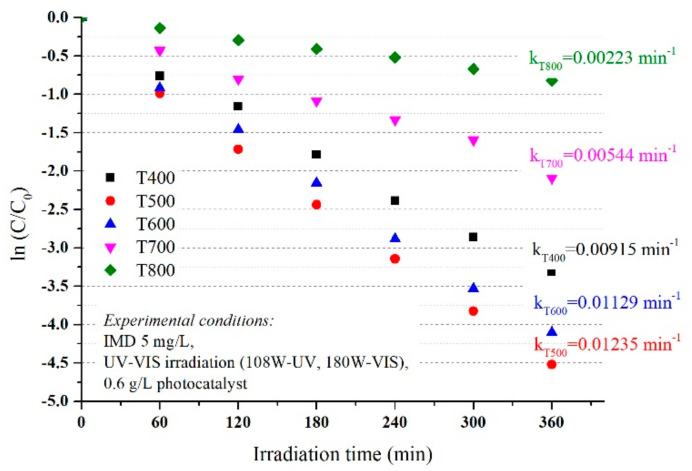
The degradation kinetics vs. catalyst type under UV–VIS simulated irradiation conditions.

The rutile content influences the values for k, k decreased from 0.01235 min^−1^ (P500, 2% rutile) to 0.01129 min^−1^ for P600 (10% rutile) down to 0.00223 min^−1^ in the P800 sample with 100% rutile (Figure 5) under UV irradiation. The values observed for reaction rate (k) under simulated visible light w not much different and decrease in the order T500 > T600 > T700 > T800 (as seen in Figure 6) with the same order of magnitude as under UV irradiation.

**Figure 7:**
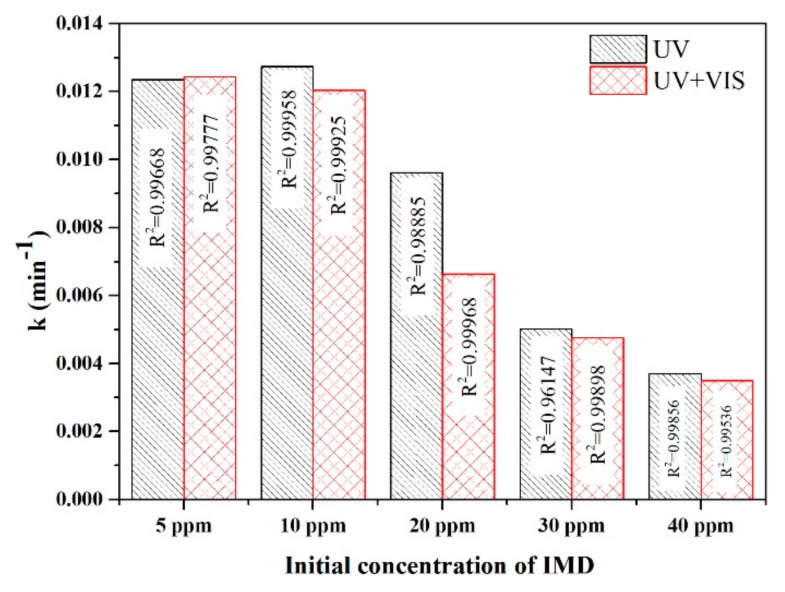
The kinetics of IMD degradation vs. irradiation and initial concentration of IMD, in the presence of the P500 photocatalyst.

The comparison between constant rate k, photon flux (irradiation), and the initial concentration of imidacloprid in the presence of the P500 photocatalyst is given in Figure 7. The first-order rate constant of imidacloprid degradation was increased from 0.0037 to 0.012 min^−1^ with the decreasing initial concentration from 40 to 10 ppm under UV irradiation and from 0.0035 to 0.012 min^−1^ under UV–VIS irradiation. The initial concentration of IMD influences the efficiency of photodegradation through the following mechanisms:

− at low IMD concentrations, the active centres on the catalyst surface are not fully occupied by the pollutant molecules, the hydroxide HO^.^ radicals responsible for the photodegradation form in higher concentrations and increases the efficiency of the photodegradation IMD; and
− at very high concentrations of IMD, the formation of hydroxide radicals on the catalyst surface is reduced because the ions adsorbed by IMD occupies the active centres.

In general, the concentrations of pesticide that reach the wastewater through the groundwater is very low, of the order of a few ppm; heterogeneous photocatalysis is an effective process for these concentrations that cannot be removed by other conventional methods such as adsorption.

### 3.3. The Scavengers Study

The reactive oxygen species (ROS) such as superoxide radicals ($O_{2}^{\cdot -}$), hydroxyl radicals (${\mathrm{HO}}^{\cdot }$), electrons ($e^{-})$, and holes ($h^{+}$) are responsible for choosing the photodegradation mechanism [7]. The effect of scavengers on imidacloprid degradation was evaluated to understand the pathway degradation and confirm the main contribution of reactive species in the photodegradation mechanism. The influence of ROS on the photodegradation mechanism was investigated at an initial concentration of 20 mg/L IMD when the photodegradation efficiencies were only 69% (UV) and 49% (simulated VIS) after 360 h of photocatalysis. The electro-hole pairs on the surface of TiO_2_ are produced by adsorbing UV photons; electron-hole pair recombination leads to a lower rate of imidacloprid photodegradation as a photocatalysis disadvantage [30]. The predominant species in the imidacloprid photodegradation mechanism in our study are electrons and superoxide radicals, as seen in Table 3, where the degradation efficiencies of IMD are up to 10% in the presence of ROS, K_2_Cr_2_O_7_ for electrons, and 1,4 benzoquinone for superoxide radicals, regardless of the radiation used, UV or a combination of UV and VIS. The electron scavengers reduces the recombination between electrons and holes and enhances the photocatalytic reaction rate of imidacloprid oxidation.

In the absence of scavengers, degradation of imidacloprid was found to be 69% in 360 min of UV irradiation and 46% under UV–VIS irradiation, respectively. However, when formic acid was used as a scavenger, the degradation of imidacloprid increased at 90% (UV) and 70% (UV–VIS), respectively (Table 3). In conclusion, in the photodegradation process of IMD, electrons play an essential role; when h^+^ scavengers were added, which allowed the electrons to react with the pollutant, it resulted in lowering the recombination rate of electron-hole charge carriers whereas with benzoquinone and potassium dichromate, the degradation was 8% and 11%, respectively. This remarkable decrease is attributed to the scavenging of $O_{2}^{\cdot -}$ and $e^{-}$, respectively. It also indicates that the most active species were $O_{2}^{\cdot -}$ electrons followed by ${\mathrm{HO}}^{\cdot }$ under both UV and UV–VIS irradiation.

### 3.4. Aquatic Toxicity of Nanoparticles and Imidacloprid on Chlorella vulgaris Growth

**Figure 8:**
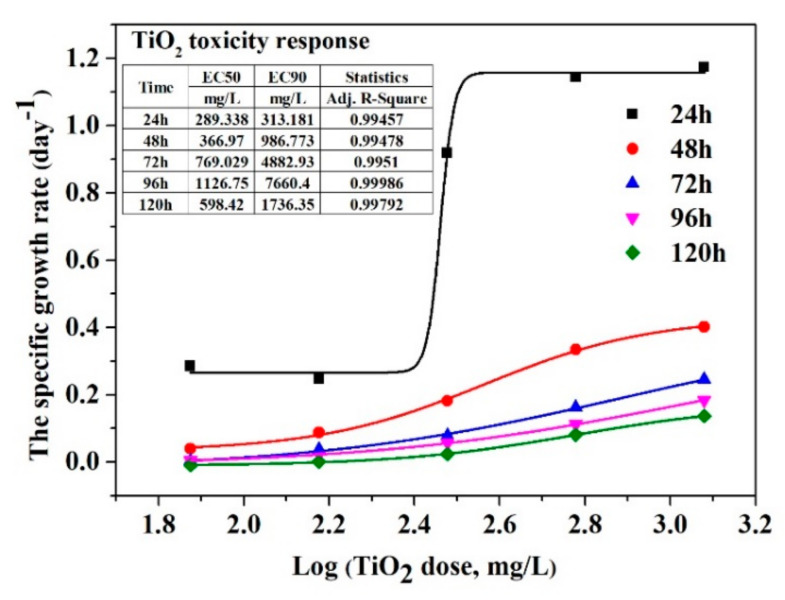
Dose-response of *Chlorella vulgaris* to TiO_2_ nanoparticles.

**Figure 9:**
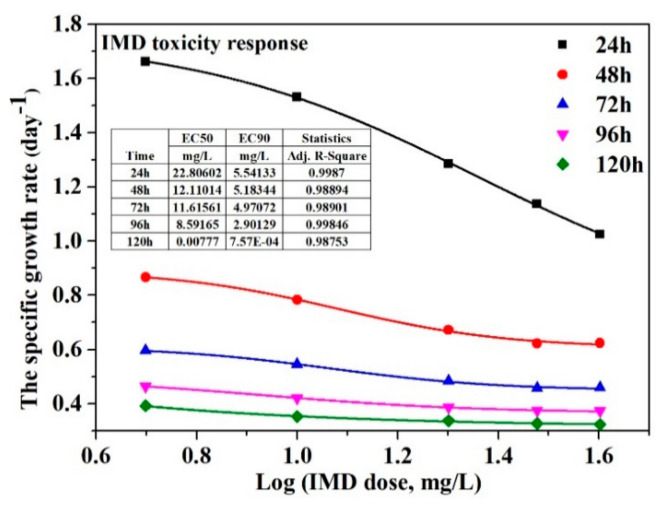
Dose-response of *Chlorella vulgaris* to imidacloprid.

Our study investigated the toxicity of TiO_2_ nanoparticles and imidacloprid on the green algae *Chlorella vulgaris* as a unicellular model organism by calculating the EC50 and EC90. The experimental concentration of TiO_2_ used in this study (150–1200 mg/L) was much higher than those presented in the literature; the choice of these working concentrations was based on the photocatalysis experiments established. Figure 8 and Figure 9 show the dose–response result in the inhibition specific growth rate of TiO_2_ nanoparticles and imidacloprid, respectively, normalised to the control. As the algal age increased, there was an increase in EC50, from 289.338 mg/L in the first 24 h to 1126.75 mg/L after 96 h algal age, followed by a decrease to 598.42 mg/L after 120 h of algae growth when the toxicant was TiO_2_ (Figure 8). The EC90 behaved similarly to EC50, increasing from 313.181 mg/L (24 h age) to 7666.4 mg/L (96 h age), then there was a decrease until 1736.36 mg/L. When IMD is an algae toxicant, a decrease of EC50 was observed from 22.8 mg/L (24 h) to 0.00777 mg/L (120 h) (Figure 9), suggesting a long-term high toxicity level when pesticides in very low concentrations are present in an aquatic environment.

## 4. Conclusions

The imidacloprid was successfully photodegradated under UV and UV–VIS irradiation when TiO_2_ prepared by sol–gel methods were used. The sol–gel route represents a promising approach toward the green and sustainable synthesis of a TiO_2_ photocatalyst for pesticide degradation. 

The first-order rate constant of imidacloprid photodegradation was increased from 0.0037 to 0.012 min^−1^ with the decreasing initial concentration from 40 to 10 ppm under UV irradiation and from 0.0035 to 0.012 min^−1^ under UV–VIS irradiation. In the photodegradation process of IMD, electrons play an essential role; the degradation efficiency of IMD increased from 69 to 90% when h^+^ scavengers were added, which allowed the electrons to react with the pollutant, resulting in lowering the recombination rate of the electron-hole charge carriers. $O_{2}^{\cdot -}$ played a significant role in the degradation mechanism, which was demonstrated with a low efficiency of 8% when 1,4 benzoquinone scavengers were added. Growth inhibition and effective concentration were determined to study the toxic effect of TiO_2_ nanoparticles and imidacloprid. The effective concentration EC50 increased from 289.338 mg/L in the first 24 h to 1126.75 mg/L after 96 h green algae age, then decreased to 598.42 mg/L after 120 h of *Chlorella vulgaris* growth when the toxicant was TiO_2_. When IMD is the aquatic toxicant, a decrease in EC50 was observed from 22.8 mg/L (24 h) to 0.00777 mg/L (120 h), suggesting a long-term high toxicity level when pesticides in very low concentrations are present in an aquatic environment.

## Figures and Tables

**Table 1:** The crystallinity, phases, and crystallite size of the investigated surfaces.

Sample	Crystallinity (%)	Phases %	2θ at (101) Anatase	Size (nm)	2θ at (110) Rutile	Size (nm)
P400	74.8	100% anatase	25.28	11.5	-	
P500	75.2	98% anatase 2% rutile	25.25	24.6	27.40	49
P600	82.2	90% anatase 10% rutile	25.24	32.4	27.39	72.5
P700	85.7	100% rutile	-	-	27.54	14.09
P800	83.5	100% rutile	-	-	27.39	10.3

**Table 2:** The texture coefficient of the investigated surfaces.

Sample	ANATASE				RUTILE			
Plane (hkl)	(101)	(004)	(200)	(204)	(110)	(101)	(111)	(220)
P400	0.6345	0.1246	0.1612	0.0796	-	-	-	-
P500	0.6525	0.0648	0.1860	0.0966	0.5132	0.2286	0.1423	0.1158
P600	0.5139	-	0.1318	0.3541	0.6831	0.0473	0.1635	0.1059
P700	-	-	-	-	0.7822	0.0365	0.1811	-
P800	-	-	-	-	0.6299	0.2298	0.0961	0.0440

**Table 3:** Removal efficiency of IMD under different scavengers involved in the radical and reaction mechanism.

Scavengers	Involved Radicals	Degradation after 6 h (%) *		Reaction Mechanism
		UV	UV–VIS	
Without scavengers	-	69	46	-
formic acid 20 mM	$h^{+}$	90	70	${\mathrm{HCOO}}^{-}+h^{+}\to {\mathrm{CO}}_{2}+H^{+}$ (8)
isopropyl alcohol 1 M	${\mathrm{HO}}^{\cdot }$	43	37	${\mathrm{HO}}^{\cdot }+{\left({\mathrm{CH}}_{3}\right)}_{2}\mathrm{CH}-\mathrm{OH}\to H_{2}O+\left({\mathrm{CH}}_{3}\right)C^{\cdot }-\mathrm{OH}$ (9)
K_2_Cr_2_O_7_ 1 mM	$e^{-}$	11	16	${\mathrm{Cr}}_{2}O_{7}^{2-}+14H^{+}+6e^{-}\to 2{\mathrm{Cr}}^{3+}+7H_{2}O$ (10)
1,4 benzoquinone 1 mM	$O_{2}^{\cdot -}$	8	8	$\mathrm{BQ}+O_{2}^{\cdot -}\to {\mathrm{BQ}}^{\cdot -}+O_{2}$ (11)

* Experimental condition: IMD initial concentration = 20 mg/L, photocatalyst: P500 with a concentration of 0.6 g/L.

## Data Availability

Not applicable.

## Supplementary Materials

The following are available online at https://www.mdpi.com/article/10.3390/nano11123197/s1, Table S1: The band-gap values of materials, Table S2: Specific surface area and pore size distribution measurments—BET, t-plot, BJH, DFT, Figure S1: The adsorption isotherms of TiO_2_ nanoparticles, Figure S2: Determining the isoelectric point of the compounds P400 (a), P500 (b), P600 (c), P700 (d), P800 (e).
