# Supplementary material for: Synthesis, Characterisation, Photocatalytic Activity, and Aquatic Toxicity Evaluation of TiO2 Nanoparticles
Source: Nanomaterials (Basel). 2021 Nov 25;11(12):3197. doi: 10.3390/nano11123197 (PMC8709270; doi:10.3390/nano11123197)
Supplement: Supplementary file 1 [file nanomaterials-11-03197-s001.zip › nanomaterials-1429829-supplementary.pdf]

Supplementary Materials

Article

Synthesis, Characterisation, Photocatalytic Activity, and Aquatic Toxicity Evaluation of TiO_2 Nanoparticles

Luminita Andronic [1,*], Alina Vladescu [2,3] and Alexandru Enesca [1]

[1] Product Design, Mechatronics and Environment Department, Transilvania University of Brasov, Eroilor 29, 500036 Brasov, Romania; a.enesca@unitbv.ro
[2] National Institute of Research and Development for Optoelectronics INOE2000, 409 Atomistilor St.,
77125 Magurele, Bucharest, Romania; alinava@inoe.ro
[3] Physical Materials Science and Composite Materials Centre, Research School of Chemistry & Applied
Biomedical Sciences, National Research Tomsk Polytechnic University, 30 Lenina Avenue, 634050 Tomsk, Russia
* Correspondence: andronic-luminita@unitbv.ro

Table 1. The band-gap values of materials.

**Samples**	**E_g (eV)**
P400	3.12
P500	3.21
P600	3.28
P700	2.96
P800	2.93

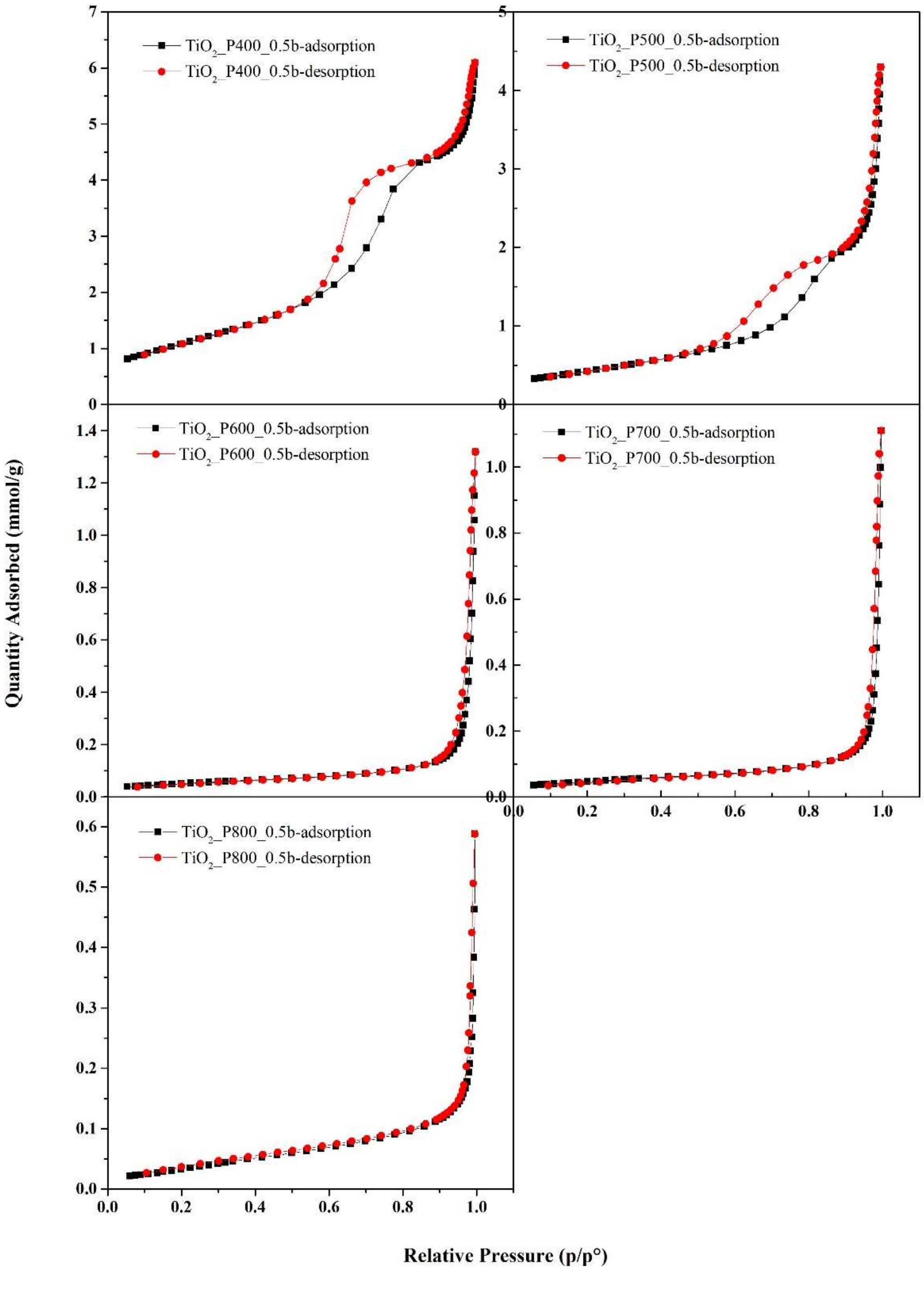

Figure 1. The adsorbtion isotherms of TiO_2 nanoparticles.

Table 2. Specific surface area and pore size distribution measurments-BET, t-plot, BJH, DFT.

Sample	BET surface area	t-plot				BJH	DFT		Nanoparticle size	IUPAC classification	
		micropore pore volume	micropore area	small micropore volume	large micropore volume	total mesopore volume	total pore volume	pore size		BDDT	Hysteresis
	m^2/g	cm^3/g	m^2/g	cm^3/g	cm^3/g	cm^3/g	cm^3/g	nm	nm		
TiO_2_P400_0.5b	89.08	0.028831	56.6736	0.010807	0.129038	0.195682	0.18916	7.32	67	IV	H2
TiO_2_P500_0.5b	34.69	0.007579	14.62	0.003436	0.03658	0.139962	0.12221	8.99	173	IV	H3
TiO_2_P600_0.5b	4.0667	0.000348	0.8318	0.000252	0.000393	0.035943	0.02803	7.05	1475	III	H1
TiO_2_P700_0.5b	3.7248	0.000257	0.7157	0.000164	0.000521	0.03008	0.02248	6.29	1610	III	H1
TiO_2_P800_0.5b	3.1262	0.000594	0.808	0.000828	0.000393	0.004909	0.00993	5.93	2484	III	H1

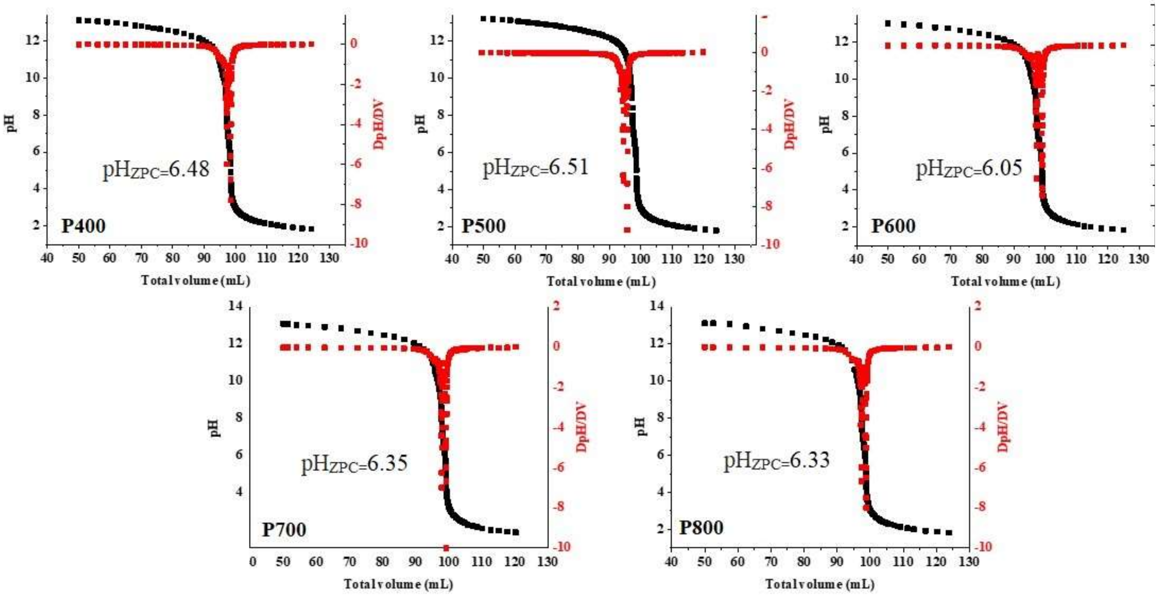

Figure 1S. Determining the isoelectric point of the compounds P400 (**a**), P500 (**b**), P600 (**c**), P700 (**d**), P800 (**e**).
